# Rab3-GEF Controls Active Zone Development at the *Drosophila* Neuromuscular Junction^1,2,3^

**DOI:** 10.1523/ENEURO.0031-16.2016

**Published:** 2016-03-12

**Authors:** Haneui Bae, Shirui Chen, John P. Roche, Minrong Ai, Chunlai Wu, Aaron Diantonio, Ethan R. Graf

**Affiliations:** 1 Department of Biology and Neuroscience Program, Amherst College, Amherst, Massachusetts 01002; 2 Department of Developmental Biology, Hope Center for Neurological Disorders, Washington University School of Medicine, St Louis, Missouri 63110; 3 Department of Cell Biology and Anatomy, Neuroscience Center of Excellence, Louisiana State University Health Sciences Center, New Orleans, Louisiana 70112

**Keywords:** Active Zone, Drosophila, Neuromuscular Junction, Rab3, Synapse

## Abstract

Synaptic signaling involves the release of neurotransmitter from presynaptic active zones (AZs). Proteins that regulate vesicle exocytosis cluster at AZs, composing the cytomatrix at the active zone (CAZ). At the *Drosophila* neuromuscular junction (NMJ), the small GTPase Rab3 controls the distribution of CAZ proteins across release sites, thereby regulating the efficacy of individual AZs. Here we identify Rab3-GEF as a second protein that acts in conjunction with Rab3 to control AZ protein composition. At *rab3-GEF* mutant NMJs, Bruchpilot (Brp) and Ca^2+^ channels are enriched at a subset of AZs, leaving the remaining sites devoid of key CAZ components in a manner that is indistinguishable from *rab3* mutant NMJs. As the *Drosophila* homologue of mammalian DENN/MADD and *Caenorhabditis elegans* AEX-3, Rab3-GEF is a guanine nucleotide exchange factor (GEF) for Rab3 that stimulates GDP to GTP exchange. Mechanistic studies reveal that although Rab3 and Rab3-GEF act within the same mechanism to control AZ development, Rab3-GEF is involved in multiple roles. We show that Rab3-GEF is required for transport of Rab3. However, the synaptic phenotype in the *rab3-GEF* mutant cannot be fully explained by defective transport and loss of GEF activity. A transgenically expressed GTP-locked variant of Rab3 accumulates at the NMJ at wild-type levels and fully rescues the *rab3* mutant but is unable to rescue the *rab3-GEF* mutant. Our results suggest that although Rab3-GEF acts upstream of Rab3 to control Rab3 localization and likely GTP-binding, it also acts downstream to regulate CAZ development, potentially as a Rab3 effector at the synapse.

## Significance Statement

The release of neurotransmitter at synapses is regulated by proteins that form the presynaptic release machine. We show here that Rab3-GEF is required for controlling the protein composition of release sites in the *Drosophila* neuromuscular junction (NMJ). Our results indicate that loss of Rab3-GEF results in the development of NMJs that are structurally and functionally indistinguishable from NMJs that lack the protein Rab3. Our studies reveal that Rab3 and Rab3-GEF act within the same molecular mechanism and support a model wherein Rab3-GEF serves as a Rab3 effector to control active zone protein composition. This work provides further insight into a novel molecular mechanism that controls the strength of synapses.

## Introduction

At synapses, neurotransmitter exocytosis is regulated by a complex of release machinery proteins. These proteins aggregate at presynaptic active zones (AZs) to form the cytomatrix at the active zone (CAZ), which controls the recruitment, docking, and priming of synaptic vesicles and enhances calcium channel accumulation (Gundelfinger and Fejtova, 2012). CAZ size and composition is variable, correlating with site-specific release efficacy. It is also plastic and can be remodeled to regulate synapse function (Sigrist and Schmitz, 2011; Michel et al., 2015). However, the mechanisms that control CAZ assembly and plasticity remain unclear.

The *Drosophila* larval neuromuscular junction (NMJ) is composed of hundreds of release sites defined by the apposition of postsynaptic clusters of glutamate receptors (GluRs) with presynaptic accumulations of the CAZ protein Bruchpilot (Brp), the fly ortholog of mammalian and *Caenorhabditis elegans* CAST/ERC/ELKS (Atwood et al., 1993; Marrus and DiAntonio, 2004; Kittel et al., 2006; Wagh et al., 2006). Several recent studies have striven to define the molecular mechanisms that control Brp aggregation at release sites. The number of AZs that contain Brp is reduced following the disruption of many proteins, including the cell adhesion proteins teneurin and neuroligin (Mosca et al., 2012), postsynaptic actin 57B (Blunk et al., 2014), the serine threonine kinase Unc-51 (Wairkar et al., 2009), the protein phosphatase PP2A (Viquez et al., 2009), and the vesicle proteins synaptotagmin-1 (Paul et al., 2015) and Rab3 (Graf et al., 2009). Of the mutants thus far identified, loss of Rab3 leads to the most severe disruption of Brp localization and distribution across AZs. At *rab3* mutant NMJs, two-thirds of AZs are devoid of essential CAZ proteins. Rather, multiple presynaptic components including Brp, calcium channels, and T-bars are concentrated at a minority of sites where they form enlarged aggregates each containing an increased number of CAZ proteins (Graf et al., 2009; Ehmann et al., 2014). This altered distribution of CAZ proteins leads to the formation of a small number of functional sites, each with enhanced release efficacy (Peled and Isacoff, 2011; Graf et al., 2009).

Rab3 is a member of a large family of small GTPases that participates in the tethering of vesicles to target membranes (Bhuin and Roy, 2014). Similar to other small GTPases, Rab3 activity is controlled by the opposing actions of GTPase accelerating proteins (GAPs) and guanine nucleotide exchange factors (GEFs) that determine its GDP- versus GTP-bound state (Cherfils and Zeghouf, 2013). Rab3 function for controlling Brp distribution requires both GTP binding and membrane association (Chen et al., 2015), but not the binding of the effector protein RIM (Rab3 interacting molecule; Graf et al., 2012). However, the mechanism by which Rab3 controls CAZ formation and the proteins with which Rab3 must interact are largely unknown.

Through a genetic screen, we now identify *Drosophila* Rab3-GEF as an essential regulator of CAZ development at the NMJ. Rab3-GEF is the orthologue of mammalian DENN/MADD/Rab3-GEP and *C. elegans* AEX-3 (Iwasaki et al., 1997; Wada et al., 1997). We find that loss of *rab3-GEF* results in the development of NMJs that are morphologically and functionally indistinguishable from *rab3* mutant NMJs. Prior work in other organisms indicates that Rab3-GEF acts upstream of Rab3 to control Rab3 activity via GDP to GTP exchange and by linking Rab3 to kinesin for proper axonal trafficking (Wada et al., 1997; Niwa et al., 2008). Consistent with these studies, we show that Rab3 trafficking is defective in the *rab3-GEF* mutant. However, these functions are not sufficient to explain the *rab3-GEF* phenotype. Transgenic expression of Rab3Q80L, a GTP-locked variant of Rab3 that rescues the *rab3* mutant, enhances the accumulation of Rab3 at *rab3-GEF* mutant NMJs but still fails to rescue the defective distribution of AZ components. Thus, in addition to its roles regulating Rab3 activity and localization, these findings support the model that Rab3-GEF also functions downstream of Rab3, potentially as a Rab3 effector protein to dock associated vesicles at active zones.

## Materials and Methods

### Fly stocks

Flies were maintained at 25°C on standard fly food. Wild-type (WT) flies were Canton S (CS) or CS outcrossed to *dvglut* ^NMJX^-*Gal4* (Daniels et al., 2008). The following fly lines were obtained from the Bloomington Stock Center: the P-element line P{EPgy2}Rab3-GEF^EY06511^, the deficiency lines Df(1)ED7289 and Df(2R)ED2076, the *UAS-cacophony-GFP* line P(UAS-cac1-EGFP)422A (Kawasaki et al., 2004), and the *UAS-YFP-rab3* line P{UASp-YFP.Rab3}4EHP^05b^ (Zhang et al., 2007). The *rab3^rup^* mutant (Graf et al., 2009) and the lines containing the *UAS-rab3* and *UAS-rab3Q80L* transgenes (Chen et al., 2015) were described previously.

### Cloning of *rab3-GEF* cDNA

To generate the 6.3 kb full-length *rab3-GEF* cDNA, we cloned two overlapping cDNA fragments spanning the entire *rab3-GEF* ORF and then sequentially ligated individual fragments together. In brief, total RNA was extracted from adult *Drosophila* heads and reverse-transcription (RT) reactions were performed using gene-specific primers to obtain two cDNA fragments: NotI-NheI and NheI-XbaI. NotI and XbaI sites were introduced into the 5′ end and 3′ end, respectively, to facilitate subcloning into the pUAST vector to generate *UAS-rab3-GEF*.

### Generation of *rab3-GEF* mutants

To generate the collection of EMS mutagenized lines, isogenic males were fed a solution of 5% sucrose and 10 mm methanesulfonic acid ethyl ester (EMS; Sigma-Aldrich) for 12–16 h. These males were mated with FM7a balanced females. <2000 F_1_ female virgin offspring were individually crossed with FM7a balanced males to create the stock collection used in the anatomical genetic screen.

Deletion of the *rab3-GEF* gene was generated by imprecise excision of the P-element P{EPgy2}Rab3-GEF^EY06511^, which is located within an intron near the 3′ end of the *rab3-GEF* gene. To initiate the excision, homozygous *y, w^-^, P{EPgy2}Rab3-GEF^EY06511^* females were crossed to *y, w^-^/Y; Xa/CyO; Δ2-3, Sb* males carrying the transposase. F_1_ male progeny *y, w^-^, P{EPgy2}Rab3-GEF^EY06511^/Y; +/CyO; +/Δ2-3, Sb* were collected and crossed to *FM7j, B^1^* females. The F_2_ progeny were screened for white-eyed females, which were crossed individually to *FM7j, B^1^/Y* males to set up stocks that were subsequently screened by PCR to identify those in which DNA surrounding the P-element had been excised. A single mutant with a substantial excision was identified, *rab3-GEF^SC225^*, and the following primer pairs were chosen to span the excised region, creating a PCR product that was sequenced to determine the precise nature of the excision: 5′-GCGTCACTTCTCCGATTCCG-3′ and 5′-CCCTTCTGGGTGAAGCACTTGCGG-3′.

### Antibody generation

Polyclonal anti sera were generated against a synthetic Rab3-GEF peptide (CDSDRELTSRRDSDQQRLH). The peptide was conjugated to KLH and injected into rabbits for the generation of antiserum (YenZym Antibodies). The antiserum was affinity purified using the peptide conjugated to SulfoLink Coupling Resin (Thermo Scientific). The antibodies were eluted from the affinity column using 0.1 m glycine, pH 2.7. The purified α-Rab3-GEF antibody was used at 1:500.

### Immunohistochemistry

Third-instar larvae were dissected in PBS and fixed in either Bouin’s fixative for 5 min or 4% formaldehyde for 20 min. Larvae were washed with PBS containing 0.1% Triton X-100 (PBT) and blocked in 5% NGS in PBT for 30 min, followed by overnight incubation in primary antibodies in 5% NGS in PBT, three washes in PBT, incubation in secondary antibodies in 5% NGS in PBT for 45 min, three final washes in PBT, and equilibration in 70% glycerol in PBS. Samples were mounted in VectaShield (Vector Laboratories). The following primary antibodies were used: mouse α-Brp, 1:250 (Developmental Studies Hybridoma Bank), rabbit α-DGluRIII, 1:2500 (Marrus et al., 2004), and rabbit α-Rab3, 1:1000. Goat Cy3-conjugated secondary antibodies against mouse and rabbit IgG and Cy3- and AlexaFluor 647-conjugated goat α-HRP were used at 1:1000 and were obtained from Jackson ImmunoResearch. AlexaFluor 488-conjugated goat secondary antibody against rabbit IgG and AlexaFluor 647-conjugated secondary antibodies against mouse and rabbit IgG were used at 1:1000 and were obtained from Life Technologies. FITC-conjugated goat α-GFP was used at 1:500 and was obtained from Abcam. Antibodies obtained from the Developmental Studies Hybridoma Bank were developed under the auspices of the National Institute of Child Health and Human Development and maintained by the Department of Biological Sciences of the University of Iowa, Iowa City, IA.

### Imaging and analysis

Samples were imaged using a Nikon C2 confocal microscope. All genotypes for an individual experiment were imaged at the same gain and set such that signals from the brightest genotype for a given experiment were not saturating. NM4b NMJs on muscle 4 were analyzed. Images were analyzed using MetaMorph software (Molecular Devices). Statistical analysis was performed using either Student’s *t* test or ANOVA followed by Bonferroni’s test for comparison of samples within an experimental group. All histograms and measurements are shown as mean±SEM. Statistical analyses are summarized in Table 1. Italicized superscript letters associated with *p* values correspond to rows in the table.

**Table 1.** Statistical Table

	Data structure	Type of test	Power (α=0.05)
a (Fig. 2B)	Normally distributed	ANOVA followed by Bonferroni’s test	1
b (Fig. 2C)	Normally distributed	ANOVA followed by Bonferroni’s test	1
c (Fig. 2E)	Normally distributed	Student’s *t* test	1
d	Normally distributed	ANOVA followed by Bonferroni’s test	1
e	Normally distributed	ANOVA followed by Bonferroni’s test	1
f	Normally distributed	Student’s *t* test	0.05241
g	Normally distributed	ANOVA followed by Bonferroni’s test	1
h	Normally distributed	ANOVA followed by Bonferroni’s test	1
i (Fig. 4B)	Normally distributed	ANOVA followed by Bonferroni’s test	1
j (Fig. 4C)	Normally distributed	ANOVA followed by Bonferroni’s test	1
k	Normally distributed	ANOVA followed by Bonferroni’s test	0.07662
l	Normally distributed	ANOVA followed by Bonferroni’s test	0.15701
m (Fig. 5B)	Normally distributed	ANOVA followed by Bonferroni’s test	1
n	Normally distributed	ANOVA followed by Bonferroni’s test	0.15085
o	Normally distributed	ANOVA followed by Bonferroni’s test	0.13093
p (Fig. 7C)	Normally distributed	Student’s *t* test	1
q (Fig. 7D)	Normally distributed	Student’s *t* test	1
r (Fig. 7D)	Normally distributed	Student’s *t* test	1
s (Fig. 7E)	Normally distributed	Student’s *t* test	1
t (Fig. 8B)	Normally distributed	ANOVA followed by Bonferroni’s test	1
u (Fig. 8D)	Normally distributed	ANOVA followed by Bonferroni’s test	1
v (Fig. 8E)	Normally distributed	ANOVA followed by Bonferroni’s test	1
w	Normally distributed	Student’s *t* test	0.05575
x	Normally distributed	Student’s *t* test	0.0937
y	Normally distributed	Student’s *t* test	1
z	Normally distributed	Student’s *t* test	0.99976
aa	Normally distributed	Student’s *t* test	0.0588
bb	Normally distributed	Student’s *t* test	0.13625

To determine the percentage of GluR clusters apposed by Brp, Brp, and DGluRIII puncta were manually counted, and DGluRIII clusters that were not opposite to a detectable Brp punctum were counted as unapposed DGluRIII clusters. MetaMorph software was used for the quantification of Brp puncta size, Cacophony-GFP cluster size, and Rab3 average intensity. For measurement of Brp area, thresholds were kept constant across all genotypes for a given experiment. Although most Brp puncta were distinct, occasional overlapping puncta were separated with the cut drawing tool. For measurements of Rab3 intensity at the NMJ, the area of the NMJ was first defined by HRP and Brp signal. For measurements of Rab3 intensity in the nerve, the area of a nerve segment was first defined by HRP signal. The average intensity of Rab3 signal within each defined NMJ or nerve segment was then calculated, and the average background intensity was subtracted.

### Electrophysiology

Two electrode voltage-clamp recordings were done in muscle six of abdominal segments A3 and A4 of wandering third instar larvae. Electrodes with resistances between 10 and 20 MΩ were used. The cells were held at −70 mV in a modified HL-3 saline solution (Stewart et al., 1994) containing the following (in mm): 70 NaCl, 5 KCl, 10 NaHCO_3_, 115 sucrose, 5 trehalose, 5 HEPES, 0.4 CaCl, and 10 MgCl. Recordings were done in voltage-clamp mode using an AxoClamp 2B Amplifier (Axon Instruments) low-pass filtered at 1kHz, and digitized at 10 kHz with an Instrutech ITC-18 computer interface using Patchmaster Software (HEKA Electronics). Cells requiring 1 nA or more holding current were discarded. Spontaneous events were recorded for 1 min and the average amplitude and frequency of the spontaneous events was quantified using Mini Analysis Software (Synaptosoft). For stimulated excitatory junctional currents (EJCs), AgCl_2_ wire in a glass suction electrode was used to stimulate the cut end of the segmental nerve for 1 ms at 1.5× the threshold voltage. A Master 8 stimulator and Isoflex stimulation isolation unit (AMPI) were used to control the duration and amplitude of the stimulation. The elicited currents from 10 successive stimulation protocols were averaged off-line using custom programs written by Josef Trapani (Amherst College) in Igor Pro software (WaveMetrics). For stimulus trains, the nerve was stimulated with 10 trains of five pulses at 20 Hz with 5 s rest intervals between trains. The 10 trains were averaged and the amplitudes of the first and fifth pulse were used to calculate the facilitation index. Histograms and measurements are shown as mean±SEM. Statistical analyses are summarized in Table 1. Italicized superscript letters associated with *p* values correspond to rows in the table.

## Results

### *rab3-GEF* mutants display defective Brp distribution

**Figure 1.**
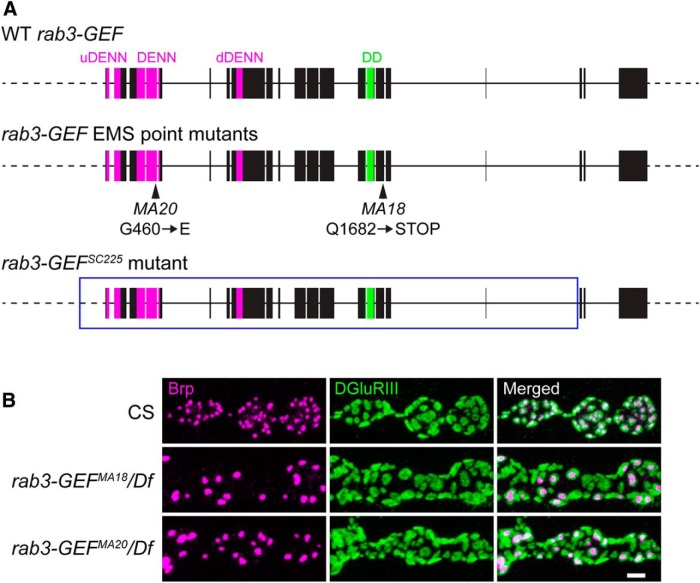
Bruchpilot distribution is disrupted in *rab3-GEF* mutant NMJs. ***A***, Schematic representations of the cloned WT *Drosophila rab3-GEF* gene, the *rab3-GEF^MA18^* and *rab3-GEF^MA20^* mutant genes generated by EMS mutagenesis, and the *rab3-GEF^SC225^* mutant gene generated by P-element excision. Magenta exonic regions correspond to the tripartite DENN domain composed of the uDENN, DENN, and dDENN modules. The green exonic region corresponds to the DD. Arrowheads identify the Q1862STOP and G460E point mutations associated with the *rab3-GEF^MA18^* and *rab3-GEF^MA20^* mutant alleles, respectively. Boxed region of the *rab3-GEF^SC225^* mutant allele denotes the part of the gene that is excised. ***B***, Confocal images of muscle 4 NMJs costained with antibodies against the presynaptic AZ protein Brp (magenta) and the postsynaptic receptor DGluRIII (green) from WT (CS), *rab3-GEF^MA18^* mutant (*rab3-GEF^MA18^*/Df(1)ED7289) and *rab3-GEF^MA20^* mutant (*rab3-GEF^MA20^*/Df(1)ED7289) third-instar larvae. Scale bar, 2 µm.

To identify proteins that control CAZ assembly and active zone composition, we conducted an anatomical genetic screen on a collection of EMS mutagenized *Drosophila* lines. A set of >2000 homozygous mutant larvae carrying mutagenized X chromosomes were dissected and costained with antibodies against the presynaptic active zone protein Brp and the essential glutamate receptor subunit DGluRIII. Immunostained NMJs were visualized by fluorescence microscopy and assessed for defects in Brp localization and distribution across active zones, including changes in Brp puncta size and number and the apposition of Brp with postsynaptic glutamate receptor clusters. From this screen, we identified two lines, MA18 and MA20, which displayed a prominent Brp distribution phenotype (Fig. 1). At *MA18* and *MA20* mutant NMJs, gross NMJ morphology and α-DGluRIII staining appears normal. However, Brp aggregates at only a subset of active zones in puncta that are significantly larger than normal, leaving the majority of GluR clusters unapposed to a Brp-positive AZ (Fig. 1*B*).

We noted that the distribution of Brp in *MA18* and *MA20* mutant NMJs was remarkably similar to the synaptic phenotype observed in *rab3* mutant larvae (Graf et al., 2009). However, *ma18* and *ma20* are not mutant alleles of *rab3* as they map to the X chromosome whereas *rab3* is on the second chromosome. To determine the gene responsible for the synaptic phenotype observed in *MA18* and *MA20* mutant NMJs, we first mapped the mutations by complementation testing. *MA18* and *MA20* are recessive alleles and transheterozygous larvae carrying both *MA18* and *MA20* failed to complement one another, suggesting that they are mutant alleles of the same gene. We identified the deficiency chromosome Df(1)ED7289 as failing to complement both *MA18* and *MA20* (Fig. 1*B*). This deficiency deletes 18 predicted genes between 13A5 and 13A12, including *rab3-GEF*, the *Drosophila* orthologue of mammalian DENN/MADD/Rab3-GEP and *C. elegans* AEX-3. In mammals and *C. elegans*, DENN/MADD/Rab3-GEP and AEX-3 are important regulators of Rab3, acting as GEFs that catalyze GDP to GTP exchange, and disruption of these genes results in defective Rab3 function (Iwasaki et al., 1997; Wada et al., 1997; Oishi et al., 1998; Coppola et al., 2002; Mahoney et al., 2006; Niwa et al., 2008). Due to the similarity between *rab3* mutant and *MA18* and *MA20* mutant NMJs, we tested whether *rab3-GEF* was responsible for the synaptic phenotype in *MA18* and *MA20* mutant larvae.

We first analyzed the structure and sequence of the *Drosophila rab3-GEF* gene. Sequence of the *rab3-GEF* transcript had been predicted by analysis of incomplete CDS clones but no full-length *rab3-GEF* cDNA had been identified. To determine experimentally the sequence of the *rab3-GEF* coding region, we cloned the gene in two overlapping cDNA fragments from mRNA extracted from adult *Drosophila* heads and ligated the two fragments together to form a 6.3 kb cDNA that spanned the entire *rab3-GEF* open reading frame. Examination of the full-length cDNA indicates that the *Drosophila rab3-GEF* transcript codes for a 2115 aa protein that contains the standard domains present in its mammalian and *C. elegans* orthologues (Schievella et al., 1997; Chow et al., 1998; Levivier et al., 2001; Marat et al., 2011), including an N-terminal tripartite DENN domain composed of the typical uDENN (upstream DENN), DENN, and dDENN (downstream DENN) modules and a single Death domain (DD) near its C-terminal end (Fig. 1*A*). *Drosophila* Rab3-GEF is ∼500–700 amino acids longer than its orthologues due to a modest lengthening of the regions separating domains and the addition of ∼300 amino acids at its C-terminal end following the Death domain.

Sequencing of *rab3-GEF* in the *MA18* and *MA20* mutant lines revealed mutations within *rab3-GEF* coding regions predicted to disrupt or eliminate important domains (Fig. 1*A*). *rab3-GEF^MA18^* contains a nonsense mutation that results in a premature stop codon in place of a glutamine residue at position 1682 downstream of the Death Domain. *rab3-GEF^MA20^* contains a missense mutation in which glycine 460 is replaced by glutamate (G460E). Residue 460 is located within the second DENN module of the tripartite DENN domain among a sequence of primarily non-polar amino acids. Although the precise functional deficits of Rab3-GEF in the *MA18* and *MA20* lines remain unknown, our analysis indicates that *rab3-GEF^MA18^* and *rab3-GEF^MA20^* are mutant alleles of *rab3-GEF*, and their lack of complementation with each other and Df(1)ED7289 suggests that the observed active zone phenotype in each is due to *rab3-GEF* dysfunction.

### *rab3-GEF^SC225^* phenocopies the active zone phenotype of the *rab3* mutant

Because the mutations present in the *rab3-GEF^MA18^* and *rab3-GEF^MA20^* alleles do not result in clear functional nulls, we generated a third *rab3-GEF* mutant by excision of a transposable P-element located within an intron near the 3′ end of the gene. Imprecise excision of P{EPgy2}Rab3-GEF^EY06511^ generated *rab3-GEF^SC225^* which contains a large excision that results in the deletion of the majority of the *rab3-GEF* gene, including the start codon and part of the predicted 5′ UTR (Fig. 1*A*). In the *rab3-GEF^SC225^* mutant, the entire coding region is removed except for the last three exons that code for the final 364 amino acids of the protein, which is unlikely to be expressed because there is no start codon. Thus, we hypothesized that *rab3-GEF^SC225^* should represent a genetic and functional null allele of *rab3-GEF* and utilized it as a basis for our subsequent studies of Rab3-GEF function.

**Figure 2.**
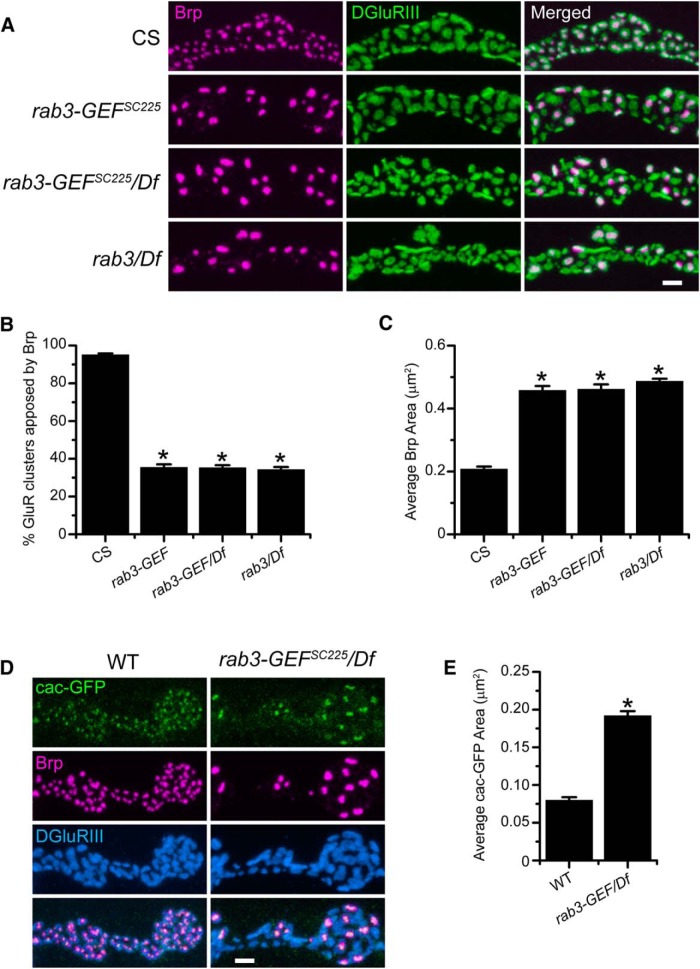
The altered distribution of Brp in *rab3-GEF^SC225^* mutant NMJs is identical to the *rab3* mutant and results in the clustering of Ca^2+^ channels at a small number of release sites. ***A***, Images of NMJs costained with α-Brp (magenta) and α-DGluRIII (green) from CS, homozygous *rab3-GEF^SC225^* mutant larvae, *rab3-GEF^SC225^*/Df(1)ED7289 mutant larvae, and *rab3^rup^*/Df(2R)ED2076 mutant larvae. Scale bar, 2 µm. ***B***, ***C***, Histograms show (***B***) the average percentage of DGluRIII clusters apposed to Brp puncta per NMJ and (***C***) the average area of individual Brp puncta for the genotypes listed in ***A***. *n* = 10 NMJs for all genotypes; (***B***) *p≪0.000001^a^ versus CS; (***C***) *p≪0.000001^b^ versus CS. ***D***, Images of NMJs expressing cacophony-GFP (green) driven by *dvglut* ^NMJX^-*Gal4* in from WT (*dvglut* ^NMJX^-*Gal4*/+; *UAS-Cacophony-GFP*/+) and *rab3-GEF^SC225^* mutant (*dvglut^NMJX^-Gal4*, *rab3-GEF^SC225^*/Df(1)ED7289; *UAS-Cacophony-GFP*/+) larvae. NMJs are costained with α-Brp (magenta) and α-DGluRIII (blue) antibodies. Scale bar, 2 µm. ***E***, Histogram shows the average area of cacophony-GFP clusters for the genotypes listed in ***D***. *n* = 10 NMJs for both genotypes; *p≪0.000001^c^ versus WT.

Initial analysis of the *MA18* and *MA20* mutants indicated that *rab3-GEF* disruption results in defective Brp distribution across active zones. Examination of Brp and DGluRIII localization in the *rab3-GEF^SC225^* mutant demonstrates that excision of the *rab3-GEF* gene also results in the concentration of Brp protein at a small fraction of available sites (Fig. 2*A*). Quantification of immunostained NMJs reveals that whereas Brp is apposed to nearly all GluR clusters at WT NMJs, in the *rab3-GEF^SC225^* mutant, only one-third of GluR clusters are apposed to a Brp punctum (Fig. 2*B*). Furthermore, average Brp punctum size is twice as large in the *rab3-GEF^SC225^* mutant compared with WT (Fig. 2*C*). In addition, Brp-GluR apposition and average Brp area are the same in both homozygous *rab3-GEF^SC225^* mutant larvae and transheterozygotes of *rab3-GEF^SC225^* and Df(1)ED7289 (Fig. 2*A*–*C*), indicating that *rab3-GEF^SC225^* behaves as a genetic null. Despite the observed active zone phenotype, *rab3-GEF^SC225^* mutants are both viable and fertile.

In mammals and *C. elegans*, loss of DENN/MADD/Rab3-GEP and AEX-3 causes defects in Rab3 function (Iwasaki et al., 1997; Yamaguchi et al., 2002; Mahoney et al., 2006; Niwa et al., 2008). To determine whether loss of Rab3-GEF mimics loss of Rab3, we compared Brp distribution between the two mutants and examined the degree of similarity (Fig. 2*A*–*C*). Quantification reveals no difference in Brp localization between *rab3*/Df and *rab3-GEF^SC225^*/Df mutants, both in terms of percentage of GluR clusters apposed to Brp (*p*=0.601*^a^*) and average Brp area (*p*=0.156*^b^*). Interestingly, *rab3-GEF^SC225^* is the first mutant identified to have a Brp distribution phenotype that is indistinguishable from the *rab3* mutant.

We also wished to quantify the severity of the Brp distribution phenotype in *rab3-GEF^MA18^* and *rab3-GEF^MA20^* mutant NMJs where the functional disruption of Rab3-GEF is less clear. Whereas the percentage of GluR clusters apposed to Brp in *rab3-GEF^MA18^* and *rab3-GEF^MA20^* mutant NMJs is significantly reduced compared with WT, the reduction is slightly less severe than in the *rab3* mutant (CS: 93.8 ± 0.9%; *rab3-GEF^MA18^/*Df(1)ED7289: 38.0 ± 1.3%; *rab3-GEF^MA20^/*Df(1)ED7289: 39.4 ± 1.6%; *rab3^rup^/*Df(2R)ED2076: 31.6 ± 1.2%; *n* = 10 NMJs for all genotypes; *p*≪0.000001*^d^* for CS vs *MA18* and *MA20*; *p*=0.00499*^d^* for *rab3* vs *MA18*; *p*=0.000393*^d^* for *rab3* vs *MA20*). Conversely, average Brp area is similar in *rab3-GEF^MA18^* and *rab3-GEF^MA20^*, and *rab3* mutant NMJs (CS: 0.19 ± 0.005 µm^2^; *rab3-GEF^MA18^/*Df(1)ED7289: 0.41 ± 0.014 µm^2^; *rab3-GEF^MA20^/*Df(1)ED7289: 0.44 ± 0.010 µm^2^; *rab3^rup^/*Df(2R)ED2076: 0.43 ± 0.011 µm^2^; *n* = 10 NMJs for all genotypes; *p*≪0.000001*^e^* for CS vs *MA18* and *MA20*; *p*=1.00*^e^* for *rab3* vs *MA18* and *MA20*). These results suggest that *rab3-GEF^MA18^* and *rab3-GEF^MA20^* behave as strong hypomorphs.

Our results indicate that Brp distribution is indistinguishable between *rab3-GEF^SC225^* and *rab3* mutant NMJs. Do other morphological similarities exist? In *rab3* mutant NMJs, multiple presynaptic components required for vesicle release are concentrated at the active zones where Brp is aggregated, including calcium channels (Graf et al., 2009). To determine whether presynaptic calcium channels are distributed similarly at *rab3-GEF^SC225^* mutant NMJs, we used the Gal4/UAS system to express a GFP-tagged version of the calcium channel subunit Cacophony in motor neurons (Fig. 2*D*). When expressed in WT neurons, cacophony-GFP accumulates opposite DGluRIII at the majority of release sites. Conversely, in *rab3-GEF^SC225^* mutant NMJs, Cacophony-GFP aggregates selectively at Brp-positive active zones, failing to accumulate opposite the majority of DGluRIII clusters (Fig. 2*D*). Furthermore, aggregations of cacophony-GFP protein are larger in the *rab3-GEF^SC225^* mutant compared with WT (Fig. 2*E*). Thus, in terms of both Brp and calcium-channel distribution, the *rab3-GEF^SC225^* mutant phenocopies the morphological defects observed at *rab3* mutant NMJs. These results are consistent with the hypothesis that Rab3 function is compromised in the *rab3-GEF^SC225^* mutant and that the developmental defects observed in the two mutants are mechanistically related.

### Rab3-GEF localizes to both the NMJ and cell body

**Figure 3.**
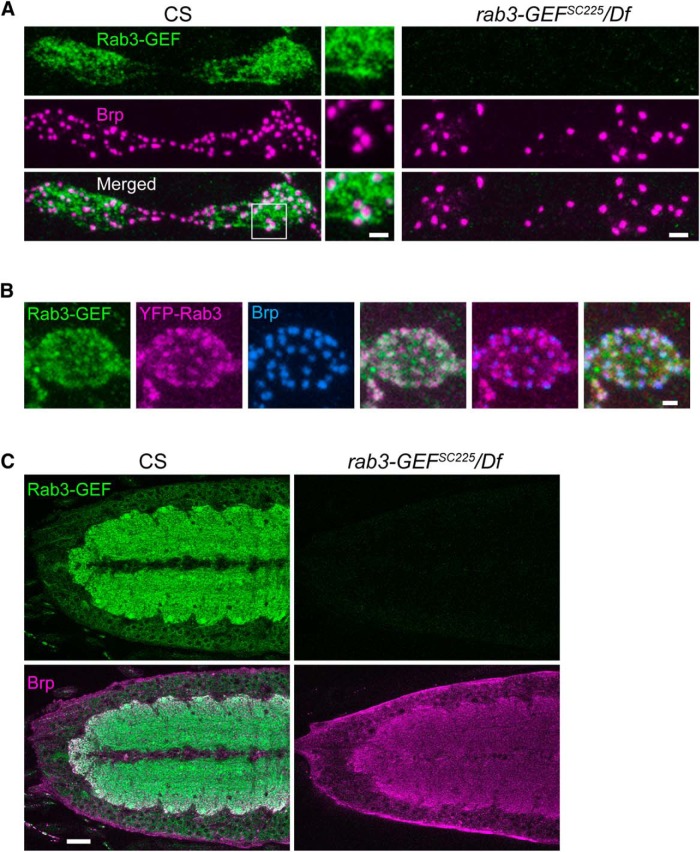
α-Rab3-GEF immunostaining reveals Rab3-GEF protein at the NMJ and in ventral nerve cord cell bodies and neuropil. ***A***, Images of NMJs immunostained with α-Rab3-GEF (green) and α-Brp (magenta) from CS and *rab3-GEF^SC225^*/Df(1)ED7289 mutant larvae. Inset, Magnified view of boxed region, indicating partial overlap of α-Rab3-GEF and α-Brp signal but little colocalization. Scale bars: lower-magnification images, 2 µm; higher-magnification inset, 1 µm. ***B***, Image of a single bouton of a WT NMJ expressing *UAS-rab3-YFP* (magenta) driven by the *dvglut^NMJX^-Gal4* driver and costained with α-Rab3-GEF (green) and α-Brp (blue). Scale bar, 1 µm. ***C***, Single optical sections of ventral nerve cords from CS and *rab3-GEF^SC225^*/Df(1)ED7289 mutant larvae immunostained with α-Rab3-GEF (green) and α-Brp (magenta). In WT brains, Rab3-GEF immunostaining is prominent in the neuropil, as well as in the surrounding neuronal cell bodies visualized by cytosolic Rab3-GEF surrounding immuno-negative nuceli. Scale bar, 20 µm.

Our morphological analysis indicates that *rab3-GEF* dysfunction results in defective AZ development. To study how Rab3-GEF functions to control synapse development, we first examined where Rab3-GEF protein localizes within *Drosophila* neurons. We generated a polyclonal antibody to a protein epitope in the region separating the uDENN and DENN modules of *Drosophila* Rab3-GEF. This antibody stains WT NMJs in a mottled pattern throughout the synaptic terminal (Fig. 3*A*). α-Rab3-GEF and α-Brp staining partially overlap, although regions of greater Rab3-GEF intensity have a tendency to be adjacent to Brp puncta rather than colocalizing with them (Fig. 3*A*, inset). Because Rab3-GEF may regulate Rab3 function, we also examined the degree of colocalization between Rab3 and Rab3-GEF at the NMJ by expressing YFP-tagged Rab3 in WT neurons with the *dvglut^NMJX^-Gal4* driver (Fig. 3*B*). Rab3-GEF and YFP-Rab3 also partially overlap, although the colocalization between YFP-Rab3 and Brp is more prominent, as described previously for endogenous Rab3 (Graf et al., 2009; Chen et al., 2015). In addition, Rab3-GEF localization at the NMJ appears generally unaffected by *rab3* disruption. The average intensity of Rab3-GEF staining at *rab3* mutant NMJs is similar to WT (CS: 100.0 ± 4.4 a.u.; *rab3/*Df: 99.1 ± 4.0 a.u.; *n* = 8 NMJs for both genotypes; *p*=0.879*^f^*).

Examination of the larval ventral nerve cord (VNC) also reveals prominent α-Rab3-GEF staining in the cell bodies and neuropil of the brain (Fig. 3*C*), suggesting that *Drosophila* Rab3-GEF may function both in neuronal cell bodies and at the synapse. Importantly, α-Rab3-GEF signal is completely absent in *rab3-GEF^SC225^* mutant animals, both at the NMJ and in the brain (Fig. 3*A*,*C*), indicating that the antibody is specific for Rab3-GEF and that Rab3-GEF protein is not expressed in the *rab3-GEF^SC225^* mutant.

We also examined Rab3-GEF protein localization in the hypomorphic *rab3-GEF^MA18^* and *rab3-GEF^MA20^* mutants. Rab3-GEF immunostaining reveals that the *MA18* point mutation results in a decrease in Rab3-GEF protein. Average Rab3-GEF intensity is significantly reduced at the NMJ (CS: 100.0 ± 6.8 a.u.; *rab3-GEF^MA18^/*Df: 5.9 ± 0.5 a.u; *n* = 8 NMJs for both genotypes; *p*≪0.000001*^g^*) and within the cell body enriched cortex of the VNC (CS: 100.0 ± 5.3 a.u.; *rab3-GEF^MA18^/*Df: 28.6 ± 2.3 a.u.; *n* = 8 single optical sections for both genotypes; *p*≪0.000001*^h^*). Conversely, in the *rab3-GEF^MA20^* mutant, Rab3-GEF staining is close to normal in VNC cell bodies (CS: 100.0 ± 5.3 a.u.; *rab3-GEF^MA20^/*Df: 84.5 ± 4.5 a.u.; *n* = 8 single optical sections for both genotypes; *p*=0.04962*^h^*) but is nearly absent at NMJs (CS: 100.0 ± 6.8 a.u.; *rab3-GEF^MA20^/*Df: 5.0 ± 0.5 a.u; *n* = 8 NMJs for both genotypes; *p*≪0.000001*^g^*). Because the *rab3-GEF^MA20^* mutant has a strong phenotype, the selective loss of Rab3-GEF at mutant NMJs but not within cell bodies suggests that there may be a local requirement for Rab3-GEF protein at the NMJ.

### Transgenic expression of *UAS-Rab3-GEF* rescues Brp distribution

**Figure 4.**
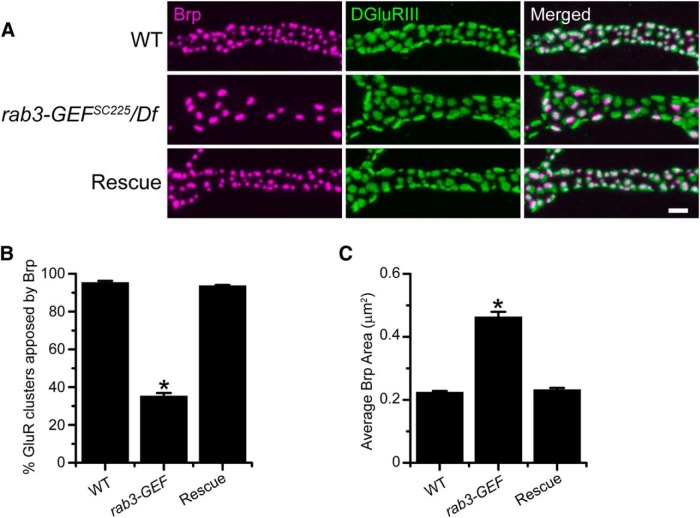
Neuronal expression of transgenic *rab3-GEF* rescues Brp distribution in *rab3-GEF^SC225^* mutant NMJs. ***A***, Images of NMJs costained with α-Brp (magenta) and α-DGluRIII (green) from WT (*dvglut^NMJX^-Gal4*/+), the *rab3-GEF^SC225^* mutant (*dvglut^NMJX^-Gal4*, *rab3-GEF^SC225^*/Df(1)ED7289), and rescue larvae corresponding to the *rab3-GEF^SC225^* mutant with neuronal expression of *UAS-rab3-GEF* (*dvglut^NMJX^-Gal4*, *rab3-GEF^SC225^*/Df(1)ED7289; *UAS-rab3-GEF*/+). Scale bar, 2 µm. ***B***, ***C***, Histograms show (***B***) the average percentage of DGluRIII clusters apposed to Brp puncta per NMJ and (***C***) the average area of individual Brp puncta for the genotypes listed in A. *n* = 11 NMJs for all genotypes; (***B***) *p≪0.000001^i^ versus both WT and rescue larvae; (***C***) *p≪0.000001^j^ versus both WT and rescue larvae.

The complete loss of Rab3-GEF protein in the *rab3-GEF^SC225^* mutant suggests that the AZ phenotype is due to the absence of *rab3-GEF*. However, to ensure that the loss of Rab3-GEF is responsible for the synaptic development defects observed in the *rab3-GEF^SC225^* mutant and to assess cell autonomy, we expressed transgenic *rab3-GEF* in the *rab3-GEF^SC225^* mutant background using the Gal4/UAS system. Neuronal expression of *UAS-rab3-GEF* by the *dvglut^NMJX^-Gal4* driver fully rescues the Brp distribution phenotype of the *rab3-GEF^SC225^* mutant (Fig. 4*A*). Whereas Brp fails to cluster at >60% of GluR clusters in the *rab3-GEF^SC225^* mutant, the percentage of GluR clusters apposed to Brp is similar to WT when *UAS-rab3-GEF* is expressed in the *rab3-GEF^SC225^* mutant (Fig. 4*B*). Transgenic expression of *UAS-rab3-GEF* also reduces average Brp puncta size to WT levels (Fig. 4*C*). Hence, neuronal expression of Rab3-GEF is required for proper AZ development.

### Rab3-GEF is required for normal short-term facilitation

Loss of Rab3-GEF results in the defective distribution of AZ components across release sites in a manner that is similar to *rab3* mutant NMJs. Because release efficacy is related to Brp accumulation (Marrus and DiAntonio, 2004; Peled and Isacoff, 2011; Peled et al., 2014), our morphological analysis suggests that *rab3-GEF^SC225^* mutant NMJs are composed primarily of very low probability, Brp-negative sites interspersed by high probability sites where Brp and calcium channels are concentrated. At *rab3* mutant NMJs, a similar distribution of AZ components results in defective release during high-frequency stimulation but normal release at low-stimulation frequencies (Graf et al., 2009). To determine the effect of *rab3-GEF* excision on NMJ function and examine how synaptic function compares between *rab3* mutant and *rab3-GEF^SC225^* mutant larvae, we performed voltage-clamp recordings from muscle 6 of segments A3 and A4 at WT, *rab3-GEF^SC225^* mutant, and *rab3* mutant NMJs. In all three genotypes, spontaneous miniature EJC amplitude is similar (WT: 0.64 ± 0.03 nA, *n* = 11; *rab3-GEF*/*Df*: 0.61 ± 0.03 nA, *n* = 13; *rab3*/*Df*: 0.66 ± 0.04 nA, *n* = 12; *p*=1.00*^k^* for *rab3-GEF* vs WT; *p*=0.829*^k^* for *rab3-GEF* vs *rab3*). Furthermore, when evoked by low-frequency stimuli in 0.4 mm Ca^2+^, EJC amplitude is comparable between the three genotypes (WT: 31.6 ± 3.0 nA, *n* = 13; *rab3-GEF*/*Df*: 38.3 ± 3.7 nA, *n* = 13; *rab3*/*Df*: 35.2 ± 5.4 nA, *n* = 12; *p*=0.744*^l^* for *rab3-GEF* vs WT; *p*=1.00*^l^* for *rab3-GEF* vs *rab3*). Thus, similar to the *rab3* mutant, at low-stimulation frequencies synaptic strength is normal at *rab3-GEF^SC225^* mutant NMJs even though the number (*n*) of AZs containing key presynaptic components is decreased.

**Figure 5.**
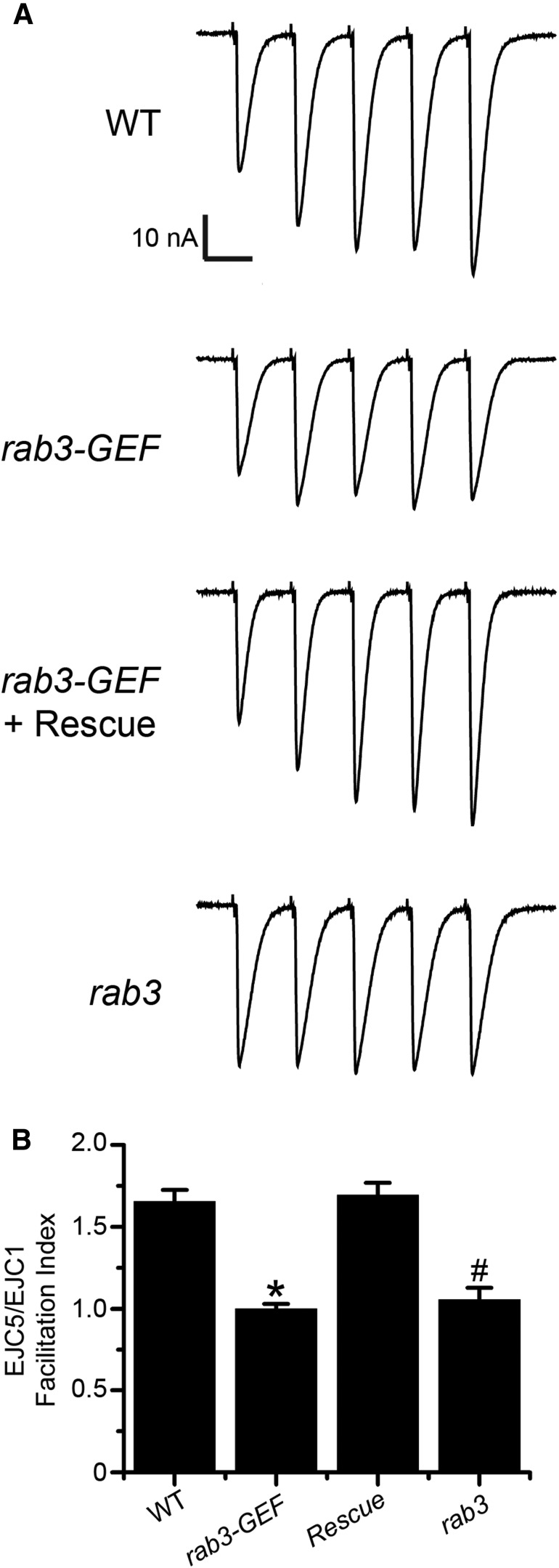
Short-term facilitation is defective in the *rab3-GEF^SC225^* mutant. ***A*** Representative EJC traces of a train of five stimuli given at a frequency of 20 Hz in 0.40 mm Ca^2+^ from WT (*dvglut^NMJX^-Gal4*/+), the *rab3-GEF^SC225^* mutant (*dvglut^NMJX^-Gal4*, *rab3-GEF^SC225^*/Df(1)ED7289), rescue larvae corresponding to the *rab3-GEF^SC225^* mutant with neuronal expression of *UAS-rab3-GEF* (*dvglut^NMJX^-Gal4*, *rab3-GEF^SC225^*/Df(1)ED7289;;*UAS-rab3-GEF*/+), and *rab3* mutant larvae (*dvglut^NMJX^-Gal4*/+; *rab3^rup^*/Df(2R)ED2076). The stimulation artifact has been removed for clarity. ***B***, Histogram showing quantification of the average FI for each of the four genotypes listed in ***A***, calculated by dividing the amplitude of the fifth EJC by the amplitude of the first EJC in a 20 Hz stimulus train. WT, *n* = 13; *rab3-GEF^SC225^* mutant, *n* = 12; *rab3-GEF^SC225^* with rescue, *n* = 14; *rab3* mutant, *n* = 9; *p≪0.000001*^m^* versus both WT and rescue larvae; #*p*=1.83 × 10^−6^ *^m^* versus WT.

In the *rab3* mutant, evoked vesicle release occurs at Brp-positive sites where the probability of release (*p*) is enhanced due to the augmented concentration of CAZ components (Graf et al., 2009; Peled and Isacoff, 2011). The increased *p* at these sites maintains neurotransmitter release despite the reduction in *n*, leading to normal EJC amplitude at low-stimulation frequencies. However, the enhanced release probability of *rab3* mutant active zones results in defective short-term facilitation (Graf et al., 2009). At WT NMJs composed primarily of lower probability release sites, high-frequency stimulation in low extracellular calcium results in facilitation. EJC size increases with each subsequent pulse, likely due to a buildup of residual calcium in the axon terminal (Zucker and Regehr, 2002). Conversely, facilitation is attenuated at NMJs with primarily high probability sites, such as is observed in the *rab3* mutant (Graf et al., 2009). Is short-term facilitation also defective in the *rab3-GEF^SC225^* mutant? To assay short-term facilitation, we stimulated WT, *rab3-GEF^SC225^* mutant, and *rab3* mutant NMJs with a short train of action potentials evoked at a frequency of 20 Hz in 0.4 mm Ca^2+^. To determine the magnitude of facilitation in each genotype, the facilitation index (FI) was calculated by dividing the amplitude of the fifth EJC by the first. Whereas facilitation is observed at WT NMJs, it is nearly absent in the *rab3-GEF^SC225^* mutant (Fig. 5). Defective facilitation in the *rab3-GEF^SC225^* mutant is equivalent to that observed in the *rab3* mutant, resulting in similar FIs between the two genotypes (*p*=0.448*^m^*). Conversely, facilitation is rescued to WT levels when the *UAS-rab3-GEF* transgene is expressed in *rab3-GEF^SC225^* mutant neurons (Fig. 5). Hence, these results suggest that the functional release sites in *rab3-GEF^SC225^* mutant NMJs have a high *p* and fire in low calcium concentrations. Additional low *p* sites are likely not recruited following subsequent stimuli, resulting in reduced facilitation. Our functional and morphological data are consistent, together indicating that *rab3-GEF^SC225^* mutant NMJs contain a reduced number of functional sites that each have a high probability of release. Furthermore, our findings reveal that loss of *rab3-GEF* phenocopies both the altered distribution of CAZ components and defective function of *rab3* mutant NMJs.

### Rab3 and Rab3-GEF act within the same pathway to control AZ development

**Figure 6.**
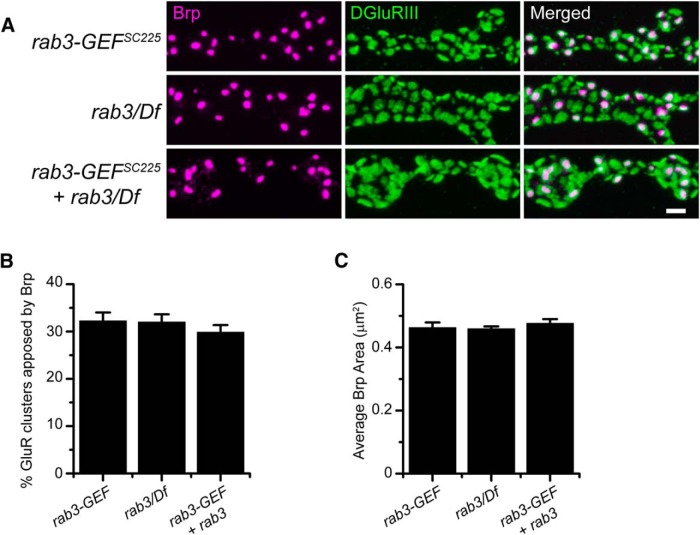
Simultaneous disruption of both *rab3* and *rab3-GEF* does not enhance the Brp distribution phenotype. ***A***, Images of NMJs costained with α-Brp (magenta) and α-DGluRIII (green) from *rab3-GEF^SC225^* mutant, *rab3^rup^*/Df(2R)ED2076 mutant, and *rab3-GEF^SC225^*; *rab3^rup^*/Df(2R)ED2076 double-mutant larvae. Scale bar, 2 µm. ***B***, ***C***, Histograms show (***B***) the average percentage of DGluRIII clusters apposed to Brp puncta per NMJ and (***C***) the average area of individual Brp puncta for the genotypes listed in ***A***. *n* = 10 NMJs for all genotypes.

Because loss of Rab3-GEF mimics the loss of Rab3, we hypothesize that the two proteins act via the same molecular pathway to control the distribution of active zone components. To test whether Rab3 and Rab3-GEF act through the same mechanism or separate, parallel pathways, we examined Brp distribution in *rab3/rab3-GEF* double-mutant larvae. If *rab3^rup^* and *rab3-GEF^SC225^* mutant alleles disrupt separate pathways, larvae mutant for both genes should display an enhanced phenotype resulting from an additive effect. Alternatively, if they act through the same pathway, disruption of both genes should result in NMJs that are identical to single knockout of each gene. Examination of α-Brp and α-DGluRIII immunostaining in *rab3/rab3-GEF* double mutant larvae reveals no difference in Brp distribution across AZs compared with *rab3* and *rab3-GEF^SC225^* single mutants (Fig. 6). The percentage of GluR clusters apposed to Brp is similar between the *rab3/rab3-GEF* double-mutant and each single mutant (*p*=0.902*^n^* for *rab3-GEF* vs double-mutant; *p*=1.00*^n^* for *rab3* vs double-mutant; Fig. 6*B*). In addition, average Brp puncta area is equivalent between all three genotypes (*p*=1.00*^°^* for *rab3-GEF* vs double-mutant; *p*=0.986*^°^* for *rab3* vs double-mutant; Fig. 6*C*). These results support the hypothesis that Rab3 and Rab3-GEF act within the same molecular pathway to control Brp distribution.

### *rab3-GEF* mutation results in defective Rab3 trafficking

How does Rab3-GEF function to control NMJ development? Prior work in mammals and *C. elegans* indicates that DENN/MADD/Rab3-GEP and AEX-3 can act upstream of Rab3 to control Rab3 function via two separate mechanisms. Orthologues of *Drosophila* Rab3-GEF catalyze GTP to GDP exchange to increase the population of active, GTP-bound Rab3 (Oishi et al., 1998; Coppola et al., 2002; Mahoney et al., 2006). In addition, DENN/MADD/Rab3-GEP and AEX-3 are required for the anterograde axonal trafficking of Rab3 from the cell body to distal synapses, acting as a linker between GTP-bound Rab3 and the kinesin family proteins KIF1A and KIF1Bβ (Iwasaki et al., 1997; Niwa et al., 2008). However, Rab3-GEF may also act downstream of Rab3 function. Studies of synaptic vesicle exocytosis in mammalian neurons indicate that DENN/MADD/Rab3-GEP acts via an unidentified postdocking mechanism to control vesicle fusion (Yamaguchi et al., 2002). Furthermore, because Rab3-GEF has been identified as a Rab3 effector during axonal trafficking, it may also act as a Rab3 effector at the AZ itself.

**Figure 7.**
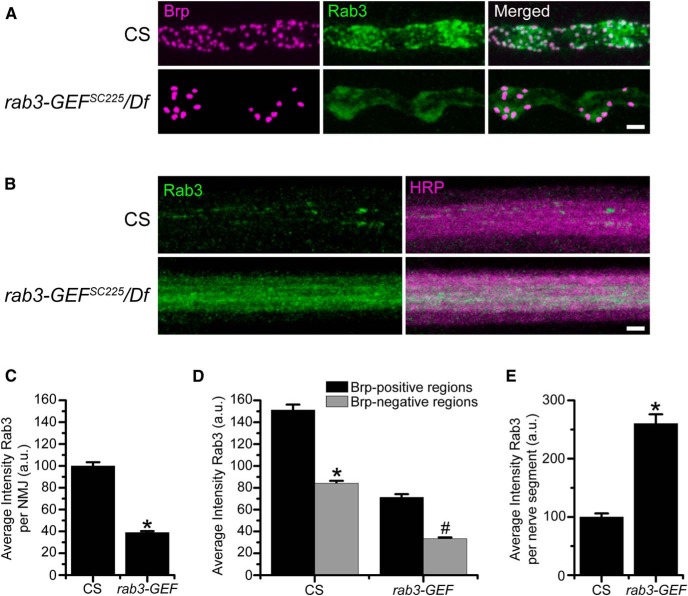
Rab3-GEF is required for proper trafficking of Rab3. ***A***, Images of NMJs costained with α-Brp (magenta) and α-Rab3 (green) from CS and *rab3-GEF^SC225^*/Df(1)ED7289 mutant larvae. Scale bar, 2 µm. ***B***, Images of motor nerve segments immunostained with α-Rab3 (green) and α-HRP (magenta) from from CS and *rab3-GEF^SC225^*/Df(1)ED7289 mutant larvae. Scale bar, 2 µm. ***C***, Histogram shows the average intensity of α-Rab3 signal throughout the entire NMJ for the genotypes listed in ***A***. *n* = 10 NMJs for both genotypes; *p≪0.000001*^p^* versus CS. ***D***, Histogram shows the average intensity of α-Rab3 signal in Brp-positive and Brp-negative regions of NMJs for the genotypes listed in ***A***. *n* = 10 NMJs for both genotypes; *p≪0.000001*^q^* versus α-Rab3 intensity at the Brp-positive regions in WT NMJs; #*p*≪0.000001*^r^* versus α-Rab3 intensity at the Brp-positive regions in *rab3-GEF* mutant NMJs. ***E***, Histogram shows the average intensity of α-Rab3 signal per nerve segment as defined by α-HRP staining for CS (*n* = 24 nerve segments) and *rab3-GEF^SC225^*/Df(1)ED7289 mutant larvae (*n* = 12 nerve segments); *p≪0.000001*^s^* versus CS.

To determine how Rab3-GEF functions in *Drosophila* neurons to control active zone protein composition, we first asked whether *Drosophila* Rab3-GEF is required for the trafficking of Rab3 to NMJs by analyzing the localization of Rab3 in the *rab3-GEF^SC225^* mutant. As described previously (Graf et al., 2009), Rab3 localizes throughout WT NMJs in a pattern suggestive of synaptic vesicle proteins but with aggregation at active zones visualized by colocalization with Brp. However, Rab3 accumulation at the NMJ is disrupted in the *rab3-GEF* mutant (Fig. 7*A*). The average intensity of α-Rab3 signal at the NMJ is reduced by approximately 65% in the *rab3-GEF^SC225^* mutant compared with WT (Fig. 7*C*). Interestingly, although the pattern of Rab3 localization at the NMJ is more diffuse in the *rab3-GEF^SC225^* mutant and Rab3 fails to aggregate in discrete puncta that colocalize with Brp, it still localizes preferentially to Brp-positive areas of the NMJ (Fig.7*A*). In both WT and *rab3-GEF^SC225^* mutant NMJs average Rab3 intensity is significantly greater when measured at Brp-positive regions compared with Brp-negative regions (Fig. 7*D*).

Reduced accumulation at the NMJ could result from defective transport of Rab3 from the cell body to the distal axon. To analyze whether axonal trafficking of Rab3 is compromised in the *rab3-GEF^SC225^* mutant, we examined the localization and intensity of α-Rab3 signal in peripheral nerves. In WT nerves, Rab3 is observed in individual axons, often localizing in discrete clusters. However, in the *rab3-GEF^SC225^* mutant, Rab3 protein accumulates throughout entire axon (Fig. 7*B*). The buildup of Rab3 in peripheral axons results in a significant increase in average Rab3 intensity per nerve segment (Fig. 7*E*). Protein aggregation in axons is a defining characteristic of defective axon transport in *Drosophila* larvae (Hurd and Saxton, 1996). Thus, the accumulation of Rab3 in peripheral nerves indicates that Rab3-GEF is required for effective transport of Rab3 in the fly.

**Figure 8.**
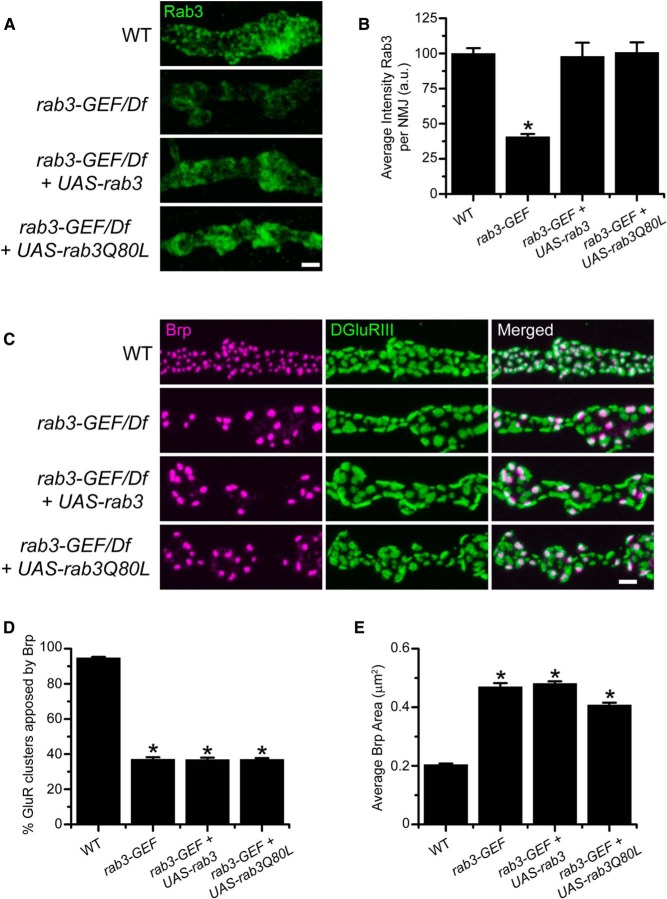
Transgenic expression of WT Rab3 or a GTP-locked variant of Rab3 increases Rab3 accumulation at the NMJ but fails to rescue Brp distribution in the *rab3-GEF^SC225^* mutant. ***A***, Images of NMJs immunostained with α-Rab3 (green) from WT (*dvglut^NMJX^-Gal4*/+), the *rab3-GEF^SC225^* mutant (*dvglut^NMJX^-Gal4*, *rab3-GEF^SC225^*/Df(1)ED7289), the *rab3-GEF^SC225^* mutant expressing *UAS-rab3* (*dvglut^NMJX^-Gal4*, *rab3-GEF^SC225^*/Df(1)ED7289;; *UAS-rab3*/+), and the *rab3-GEF^SC225^* mutant expressing *UAS-rab3Q80L* (*dvglut^NMJX^-Gal4*, *rab3-GEF^SC225^*/Df(1)ED7289;; *UAS-rab3Q80L*/+). Scale bar, 2 µm. ***B***, Histogram shows the average intensity of α-Rab3 signal throughout the entire NMJ for the genotypes listed in ***A***. *n* = 10 NMJs for all genotypes; *p= 6.7 × 10^−7^*^t^* versus WT, 1.35 × 10^−6^*^t^* versus the *rab3-GEF* mutant expressing *UAS-rab3*, and 5.12 × 10^−7^*^t^* versus the *rab3-GEF* mutant expressing *UAS-rab3Q80L*. ***C***, Images of NMJs costained with α-Brp (magenta) and α-DGluRIII (green) from the genotypes listed in ***A***. Scale bar, 2 µm. ***D***, ***E***, Histograms show (***D***) the average percentage of DGluRIII clusters apposed to Brp puncta per NMJ and (***E***) the average area of individual Brp puncta for the genotypes listed in ***A***. *n* = 10 NMJs for all genotypes; (***D***) *p≪0.000001*^u^* versus WT; (***E***) *p≪0.000001*^v^* versus WT.

We next tested whether defective Brp distribution results simply from reduced levels of Rab3 protein at the NMJ. Even though the axonal transport of Rab3 is compromised in the *rab3-GEF^SC225^* mutant, Rab3 protein still accumulates at mutant NMJs, albeit at lower levels (Fig. 7), indicating that Rab3 is able to reach the axon terminal in the absence of Rab3-GEF. Therefore, to increase Rab3 protein levels at the NMJ, we enhanced *rab3* expression, using the *dvglut^NMJX^-Gal4* neuronal driver to express WT transgenic *UAS-rab3* in the *rab3-GEF^SC225^* mutant background. Following transgenic expression of *UAS-rab3*, Rab3 protein levels in the *rab3-GEF^SC225^* mutant are significantly increased (Fig. 8*A*). Average intensity of α-Rab3 signal at the NMJ is equivalent between WT NMJs and *rab3-GEF^SC225^* mutant NMJs expressing *UAS-rab3* (Fig. 8*A*,*B*). However, increased Rab3 expression does not rescue Brp distribution in *rab3-GEF^SC225^* mutant NMJs (Fig. 8*C*). Analysis of α-Brp and α-DGluRIII staining in *rab3-GEF^SC225^* mutant larvae reveals that *UAS-rab3* expression has no effect on the percentage of GluR clusters apposed to Brp or average Brp puncta area (Fig. 8*D*,*E*). Thus, defective AZ development in the *rab3-GEF^SC225^* mutant is not solely due to the lack of Rab3 protein at the NMJ.

### Expression of a GTP-locked variant of Rab3 fails to rescue *rab3-GEF^SC225^* mutant NMJs

Because overexpression of WT Rab3 fails to rescue the synaptic phenotype of *rab3-GEF^SC225^* mutant NMJS, Rab3-GEF must have additional required functions for active zone development. Enhanced expression of WT Rab3 may not rescue AZ development due to defective guanine nucleotide cycling of both endogenous and transgenically expressed Rab3. Rab3 cycles between an active GTP-bound state and an inactive GDP-bound state. Following the binding of GTP, Rab3 hydrolyzes the guanine nucleotide to GDP, inactivating itself in a process that is regulated by Rab3-GAP (GTPase accelerating protein; Cherfils and Zeghouf, 2013). GEF proteins act as exchange factors that catalyze the release of GDP so that a new GTP molecule can bind (Bos et al., 2007). Hence, defective GEF activity in *rab3-GEF^SC225^* mutant neurons may result in a buildup of GDP-bound Rab3 and a comparable reduction in the GTP-bound population due to an inability of Rab3 to release GDP following hydrolysis.

Because Rab3 must be GTP-bound to control Brp distribution across active zones (Chen et al., 2015), defective guanine nucleotide exchange in the *rab3-GEF^SC225^* mutant may lead to the observed synaptic phenotype. Therefore, we tested whether expression of Rab3Q80L, a GTP-locked variant of Rab3, could rescue Brp distribution in *rab3-GEF^SC225^* mutant neurons. The Q80L mutation inhibits the GTPase activity of Rab3 even in the presence of Rab3-GAP, resulting in a constitutively active variant (Brondyk et al., 1993). We previously showed that expression of Rab3Q80L rescues both the morphological and functional deficits of *rab3* mutant NMJs, indicating that it is functional in the presence of Rab3-GEF (Chen et al., 2015). To determine whether GTP-locked Rab3 rescues *rab3-GEF* mutant NMJs, we assayed Brp distribution in *rab3-GEF^SC225^* mutant neurons expressing *UAS-rab3Q80L*. Driving *UAS-rab3Q80L* with *DVgult-Gal4* in *rab3-GEF^SC225^* mutant larvae increases the accumulation of Rab3 protein at the NMJ (Fig. 8*A*). Average intensity of α-Rab3 staining at the NMJ is similar between WT animals and *rab3-GEF^SC225^* mutant larvae expressing either the *UAS-rab3* or *UAS-rab3Q80L* variants of transgenic Rab3 (Fig. 8*B*). However, expression of *UAS-rab3Q80L* fails to rescue the Brp-GluR apposition phenotype of the *rab3-GEF^SC225^* mutant (Fig. 8*C*). The percentage of GluR clusters apposed to Brp does not increase following *UAS-rab3Q80L* expression in the *rab3-GEF^SC225^* mutant (Fig. 8*D*). Interestingly, driving transgenic Rab3Q80L in the *rab3-GEF^SC225^* mutant does modestly but significantly decrease average Brp puncta area (*p*=3.07 × 10^−5^*^v^*). Although this may correspond to a mild rescue of Brp puncta size, previous analysis indicates that Rab3Q80L expression also reduces average Brp puncta area when driven in WT NMJs (Chen et al., 2015), suggesting that Rab3Q80L may have a gain of function affect that decreases Brp cluster size regardless of the presence or absence of Rab3-GEF.

It is possible that Rab3-GEF is required for Rab3Q80L to bind GTP. While this may explain the inability of Rab3Q80L to rescue Brp distribution in the *rab3-GEF^SC225^* null mutant, we would predict that Rab3Q80L should be GTP-bound when driven in the *rab3-GEF^MA18^* and *rab3-GEF^MA20^* hypomorphic alleles that have unaltered death domains and retain partial function. However, similar to our analysis of *rab3-GEF^SC225^* mutant NMJs, neuronal expression of *UAS-rab3Q80L* fails to rescue the percentage of GluR clusters apposed to Brp when driven in either the *rab3-GEF^MA18^* mutant (*rab3-GEF^MA18^/*Df: 36.1 ± 0.7%; *UAS-rab3Q80L* expressed in *rab3-GEF^MA18^*/Df: 35.7 ± 1.4%: *n* = 8 NMJs for both genotypes; *p*=0.814*^w^*) or the *rab3-GEF^MA20^* mutant (*rab3-GEF^MA20^/*Df: 40.1 ± 2.0%; *UAS-rab3Q80L* expressed in *rab3-GEF^MA18^*/Df: 38.3 ± 1.8%; *n* = 8 NMJs for both genotypes; *p*=0.523*^x^*). Also similar to the *rab3-GEF^SC225^* mutant, a moderate decrease is observed in average Brp puncta size following Rab3Q80L expression in the the *rab3-GEF^MA18^* mutant (*rab3-GEF^MA18^/*Df: 0.44 ± 0.007 µm^2^; *UAS-rab3Q80L* expressed in *rab3-GEF^MA18^/*Df: 0.32 ± 0.010 µm^2^; *n* = 8 NMJs for both genotypes; *p*=1.17 × 10^−7^*^y^*) and in the *rab3-GEF^MA20^* mutant (*rab3-GEF^MA20^/*Df: 0.42 ± 0.011 µm^2^; *UAS-rab3Q80L* expressed in *rab3-GEF^MA18^/*Df: 0.34 ± 0.009 µm^2^; *n* = 8 NMJs for both genotypes; *p*=3.96 × 10^−5^*^z^*). Hence, Rab3Q80L is unable to rescue the *rab3-GEF* apposition phenotype even in the presence of genetic hypomoprhs of Rab3-GEF that retain some activity. Because Rab3-GEF levels are significantly reduced at *rab3-GEF^MA18^* and *rab3-GEF^MA20^* mutant NMJs, it is possible that GEF activity is required locally at the NMJ to maintain the GTP-bound state of Rab3Q80L. However, Rab3-GEF protein is present in the cell bodies of *rab3-GEF^MA18^* and *rab3-GEF^MA20^* mutant neurons at reduced or nearly normal levels, respectively, and may thus be available to convert Rab3Q80L to a GTP-bound state prior to being trafficked to the NMJ.

Together, these results indicate that even though expression of Rab3Q80L increases Rab3 accumulation at the NMJ and should enhance the population of GTP-bound Rab3, it is unable to rescue Brp distribution in multiple mutant alleles of *rab3-GEF*. Thus, our mechanistic studies suggest that although Rab3-GEF may act upstream of Rab3 to control Rab3 trafficking and activation in *Drosophila* neurons, it may also play an additional role that is downstream of Rab3. Because the *rab3* and *rab3-GEF* mutant phenotypes are identical, this additional function is likely associated with Rab3 itself. It also likely requires the combined action of both Rab3 and Rab3-GEF as enhancing Rab3-GEF expression in the *rab3* mutant fails to rescue either the percentage of GluR clusters apposed to Brp (*rab3* mutant: 33.7 ± 0.9%; *UAS-rab3-GEF* expressed in *rab3* mutant: 34.2 ± 1.1%; *n* = 10 NMJs for both genotypes; *p*=0.77393*^aa^*) or average Brp area (*rab3* mutant: 0.39 ± 0.005 µm^2^; *UAS-rab3-GEF* expressed in *rab3* mutant: 0.38 ± 0.015 µm^2^; *n* = 10 NMJs for both genotypes; *p*=0.381*^bb^*). We hypothesize that Rab3-GEF may act as an effector of Rab3 in the process of docking Rab3-associated vesicles at release sites to control protein composition at AZs.

## Discussion

We show that Rab3-GEF controls CAZ assembly and the distribution of AZ components across release sites at the *Drosophila* NMJ. In the *rab3-GEF^SC225^* mutant, Brp and calcium channels fail to cluster at two-thirds of available sites. Rather, CAZ components are concentrated at a minority of sites where they aggregate in enlarged clusters. *rab3-GEF^SC225^* mutant NMJs function normally when exposed to low-stimulation frequencies. However, high-frequency stimulation reveals defective short-term facilitation in the *rab3-GEF^SC225^* mutant, a hallmark of NMJs composed primarily of sites exhibiting high-release probability characteristics. Thus, both morphological and functional studies suggest that *rab3-GEF* disruption results in the formation of a small number of functional sites with enhanced release efficacy.*rab3-GEF^SC225^* mutant NMJs have a synaptic phenotype that is indistinguishable from NMJs in the *rab3* mutant (Graf et al., 2009), suggesting that they function together to control AZ composition. Prior studies of the mammalian and *C. elegans* orthologues DENN/MADD/Rab3-GEP/AEX-3 indicate that *Drosophila* Rab3-GEF likely functions as a guanine nucleotide exchange factor for Rab3, as well as a Rab3 effector that participates in the axonal transport of Rab3 to synapses (Iwasaki et al., 1997; Wada et al., 1997; Oishi et al., 1998; Coppola et al., 2002; Mahoney et al., 2006; Niwa et al., 2008). Therefore, it is possible that Rab3 itself is dysfunctional in the mutant. In support of this, we show that loss of Rab3-GEF results in defective trafficking of Rab3 and reduced accumulation of Rab3 at NMJs. However, mechanistic studies reveal that the *rab3-GEF* synaptic phenotype cannot be fully explained by loss of Rab3 function. Attempts to rescue the *rab3-GEF^SC225^* synaptic phenotype through transgenic expression of Rab3 to enhance its accumulation at the NMJ fail to rescue Brp distribution, potentially due to loss of GEF activity and a reduction in GTP-bound Rab3 in the *rab3-GEF* mutant. However, expression of Rab3Q80L, a GTP-locked variant, also fails to rescue the *rab3-GEF^SC225^* mutant phenotype even though it should enhance the GTP-bound population of Rab3 and suppress the effects caused by loss of GEF activity. Thus, our results support the model that in addition to its roles in Rab3 transport and guanine nucleotide exchange, Rab3-GEF is also required downstream of Rab3 function. We hypothesize that Rab3-GEF acts as a Rab3 effector to dock Rab3-associated vesicles at the synapse for proper CAZ assembly.

### Control of CAZ assembly by Rab3

Double-knockout of both *rab3* and *rab3-GEF* fails to enhance the NMJ phenotype, indicating that the two proteins likely act within the same molecular pathway to control AZ development. How does Rab3 control CAZ assembly? Prior mutational studies suggest that *Drosophila* Rab3 acts to localize CAZ proteins via a mechanism that is typical of Rab proteins. GTP-binding and residues required for effector interaction and membrane association are essential for proper Rab3 function at the NMJ (Chen et al., 2015). The requirement of the Rab3 C-terminal membrane association domain suggests that Rab3 may control Brp distribution via a standard vesicle-docking mechanism. However, RIM is not required for Rab3 to control Brp distribution (Graf et al., 2012). Both the type of vesicle and the effector proteins involved in its tethering to target membranes remain unknown.

Mammalian Rab3 is associated with neurotransmitter-filled synaptic vesicles, Piccolo–Bassoon transport vesicles (PTVs), and other types of secretory vesicles (Darchen and Goud, 2000; Schlüter et al., 2002; Shapira et al., 2003). The role of Rab3 in synaptic vesicle dynamics has been extensively studied (Wang et al., 1997; Schlüter et al., 2006). We have previously shown that general alterations in synaptic vesicle release caused by increasing or decreasing neuronal excitability do not affect Brp distribution in WT or *rab3* mutant NMJs (Graf et al., 2009), so it is unlikely that Rab3 controls Brp distribution via a synaptic vesicle associated mechanism. Interestingly, recent studies of the synaptic vesicle protein synaptotagmin-1 (Syt) reveal that Syt disruption also results in Brp localization defects, although the morphological phenotype resulting from Syt knockdown is different than that observed in *rab3* and *rab3-GEF* mutant NMJs (Paul et al., 2015). The fact that both Rab3 and Syt are required for proper Brp localization suggests that a linkage may exist between neurotransmitter release and CAZ assembly; however, the mechanism of this potential linkage is unclear. A simpler explanation for Rab3 function in the control of CAZ assembly involves the docking of transport vesicles homologous to mammalian PTVs, which contain a pre-assembled complex of AZ proteins for insertion at nascent release sites during synapse development (Zhai et al., 2001). Such a model is consistent with previous studies that suggest that *Drosophila* Rab3 acts to nucleate the formation of new Brp clusters at AZs (Graf et al., 2009). However, an analogous population of transport vesicles has not been identified in *Drosophila* neurons, and further work will be required to determine whether such vesicles are involved in this mechanism of Rab3 action to control Brp distribution.

### Analysis of Rab3-GEF function for controlling AZ composition

We now show that Rab3-GEF is an essential player in this Rab3-associated mechanism to control CAZ assembly. Previous work in other organisms indicates that Rab3-GEF acts upstream of Rab3 to regulate Rab3 function. Consistent with studies in mammals and *C. elegans* (Iwasaki et al., 1997; Niwa et al., 2008), we show that *Drosophila* Rab3-GEF is required for the axonal trafficking of Rab3. Rab3 protein accumulates in axon shafts, leading to decreased levels at *rab3-GEF^SC225^* mutant NMJs. Disrupted localization of Rab3 and its associated vesicles may lead to defective Brp distribution in the *rab3-GEF^SC225^* mutant. However, enhanced expression of both transgenic WT Rab3 and Rab3Q80L fail to rescue the *rab3-GEF^SC225^* mutant phenotype even though the accumulation of Rab3 is increased at NMJs to endogenous levels. These results suggest that disrupted Brp distribution in the *rab3-GEF^SC225^* mutant is not solely due to defective Rab3 trafficking. Conversely, although enhanced expression of Rab3 may increase the overall accumulation of Rab3 at the terminal, Rab3-GEF may also be required for more discrete localization of Rab3 within the NMJ, which may not be rescued by Rab3 overexpression. The localization pattern of Rab3 within the NMJ in *rab3-GEF^SC225^* mutants expressing transgenic Rab3 differs from endogenous Rab3; however, this may also be due to the fact that CAZ protein distribution is defective at these NMJs.

A second potential mechanism of Rab3-GEF action is via its role as a GEF. The mammalian and *C. elegans* orthologues of *Drosophila* Rab3-GEF act as GEFs to catalyze GDP to GTP exchange (Wada et al., 1997; Mahoney et al., 2006). It has previously been shown that GTP-binding is required for Rab3 to control Brp distribution as GTP-binding defective variants of Rab3 fail to rescue the *rab3* mutant phenotype (Chen et al., 2015). Thus, *rab3-GEF* knockout may disrupt Rab3 function in *Drosophila* neurons by reducing the GTP-bound population of Rab3. However, our studies indicate that the *rab3-GEF* synaptic phenotype cannot solely be due to loss of GEF activity or defective Rab3 localization. Enhancing the population of GTP-bound Rab3 by expression of Rab3Q80L, a hydrolysis defective variant, fails to rescue the *rab3-GEF^SC225^* mutant phenotype. Rab3Q80L accumulates at the NMJ at levels similar to endogenous Rab3. Furthermore, previous analysis indicates that Rab3Q80L can rescue the morphological and physiological phenotypes of the *rab3* mutant, indicating that it is functional when Rab3-GEF is present. Hence, the lack of rescue following Rab3Q80L expression in the *rab3-GEF* mutant suggests that the synaptic phenotype is not simply due to loss of GEF activity. Additional lines of evidence support this finding. GEF activity has been mapped to the death domain in the mammalian and *C. elegans* orthologues of Rab3-GEF. However, *rab3-GEF^MA18^* and *rab3-GEF^MA20^* mutants both have synaptic phenotypes nearly as severe as the *rab3-GEF^SC225^* mutant, even though the death domain is intact in the *MA18* and *MA20* mutant alleles. Furthermore, studies in mice reveal the presence of significant amounts of GTP-bound Rab3 despite DENN/MADD disruption (Yamaguchi et al., 2002; Niwa et al., 2008), potentially due to the presence of other proteins that can act as GEFs for Rab3.

It is possible that Rab3Q80L itself requires the nucleotide exchange activity of Rab3-GEF to transition into a GTP-bound state, which could explain the lack of rescue when Rab3Q80L is expressed in the *rab3-GEF^SC225^* mutant. However, Rab3Q80L also fails to rescue the synaptic phenotype when driven in the *rab3-GEF^MA18^* and *rab3-GEF^MA20^* hypomorphs that retain some activity. This may be due to a general decrease in Rab3-GEF protein throughout *rab3-GEF^MA18^* mutant neurons. However, Rab3-GEF protein is present at nearly normal levels in the cell bodies of *rab3-GEF^MA20^* mutant neurons even though it is absent at *rab3-GEF^MA20^* mutant NMJs. Although it is possible that Rab3Q80L requires GEF activity in the NMJ to maintain its active state, the presence of Rab3-GEF in *rab3-GEF^MA20^* cell bodies suggests that activation of Rab3Q80L in the *rab3-GEF^MA20^* mutant could occur prior to trafficking to the NMJ. In mammalian neurons, axonal transport of Rab3 is most effective when Rab3 is in a GTP-bound state (Niwa et al., 2008), suggesting that GEF activity generally occurs in the cell body, as well as at the synapse. Thus, together, our results suggest that Rab3-GEF plays other roles in the control of AZ development in addition to its functions in Rab3 trafficking and as a GEF.

How might Rab3-GEF act in addition to its previously identified roles? Because the *rab3-GEF* and *rab3* mutant phenotypes are indistinguishable from each other and from the double-mutant, this additional function must be within the Rab3 pathway. We hypothesize that Rab3-GEF also acts downstream of Rab3 to control AZ composition. Rab3-GEF is a Rab3 effector protein, linking Rab3 to kinesin for axonal trafficking (Niwa et al., 2008). Rab3-GEF may also act as a Rab3 effector at the NMJ itself, playing a direct role in the docking of Rab3-associated vesicles at the plasma membrane. Conversely, Rab3-GEF may be required for an unidentified postdocking step as has been suggested by studies of synaptic vesicle exocytosis in mouse neurons lacking Rab3-GEP (Yamaguchi et al., 2002). Additional work will be required to determine the mechanism by which Rab3 and Rab3-GEF act to control CAZ protein composition at active zones.

## Synthesis

The decision was a result of the Reviewing Editor Vivian Budnik and the peer reviewers coming together and discussing their recommendations until a consensus was reached. A fact-based synthesis statement explaining their decision and outlining what is needed to prepare a revision is listed below. The following reviewers agreed to reveal their identity: J. Troy Littleton, Dion Dickman

The authors have addressed prior comments by reviewers.

While no additional experiments are required, authors might consider suggestions by reviewer 2.

Reviewer 1

The authors have addressed my prior concerns by showing Rab3-GEF localization and levels in the mutants, an the localizaiton of Rab3-GEF in rab3 mutants. The lack of rescue of the Rab3 mutant phenotype by Rab3-GEF is also a nice addition. Overall, a solid study of a new player in Rab3 active zone biology.

Reviewer 2

The authors conduct a rigorous genetic analysis of Rab3-GEF function in Drosophila, elegantly demonstrating that mutations in Rab3-GEF effectively phenocopy mutants in Rab3. This point is rigorously shown and makes up the bulk of the study. While this discovery itself is not necessarily surprising, the thoroughness of their analysis, clarity of their findings, and importance in understanding this layer of regulation (transport and regulation of Rab3 by Rab3-GEF) should be of considerable interest to the field.

Important questions remain regarding the mechanistic relationship between Rab3 and Rab3-GEF, and the enigma of why a constitutively active Rab3 does not rescue Rab3-GEF mutants. The authors do a commendable job of attempting to provide insight into these difficult questions, proposing the idea that in addition to Rab3-GEF regulating Rab3 activity, it also may have separate roles in active zone regulation, perhaps "downstream" of Rab3.

The authors have already performed a lot of the obvious experiments to provide insight into these questions, so I would not demand that additional ones, as most would require work that itself would be an entire new study. However, it would be of interest to know the answer to these experiments:

1. The authors have already done the rescue experiments of overexpressing Rab3 and constitutively active Rab3 in Rab3GEF mutant backgrounds and did not see rescue. However, have the authors attempted to rescue active zone organization by overexpressing Rab3-GEF in a Rab3 mutant alone and perhaps overexpressing both Rab3-GEF and constitutively active Rab3 in Rab3 mutants? This may provide insight into whether Rab3 expression or activity is needed for the proposed downstream requirements of Rab3-GEF.

2. Another open question is whether the GTP exchange activity of Rab3-GEF is needed for its functions in Rab3 transport and/or downstream functions in active zone organization. Although some insights are made by using the MA20 allele, a more specific way to address this would be to specifically mutate the GEF activity in a Rab3-GEF cDNA and then attempt to rescue Rab3-GEF mutants (and monitor Rab3 transport with and without expression of constitutively active Rab3) with this transgene.

Nonetheless, this study raises interested questions about the role and regulation of Rab3 and Rab3-GEF that should be of interest to many readers.
